# Safety, pharmacokinetic and pharmacodynamic properties of TV-1106, a long-acting GH treatment for GH deficiency

**DOI:** 10.1530/EJE-15-0554

**Published:** 2015-11

**Authors:** Orit Cohen-Barak, Anat Sakov, Michele Rasamoelisolo, Merav Bassan, Kurt Brown, Boaz Mendzelevski, Ofer Spiegelstein

**Affiliations:** 1 Teva Pharmaceuticals, Inc., 12 Hatrufa Street, Netanya 42504, Israel; 1 Teva Biopharmaceuticals, Inc., Rockville, Maryland, USA; 2 Teva Pharmaceuticals, Inc., Frazer, Pennsylvania, USA; 3 Cardiac Safety Consultants Ltd, London, UK

## Abstract

**Background:**

TV-1106 (Teva Pharmaceuticals) is a genetically fused recombinant protein of human GH (hGH) and human serum albumin, in development for treatment of GH deficiency (GHD). TV-1106 is expected to have an extended duration of action compared to daily GH treatment and may enable a reduction in the frequency of injections and improve compliance and quality of life for adults and children requiring GHD therapy.

**Objective:**

To assess the safety, local tolerability, pharmacokinetics and pharmacodynamics of TV-1106 following single s.c. injections in healthy male volunteers.

**Methods:**

Subjects (*n*=56) were assigned to one of seven ascending dose groups (3–100 mg) and received either a single dose of TV-1106 (*n*=6) or placebo (*n*=2) by s.c. injection.

**Results:**

Eighteen subjects reported 43 adverse effects (AEs), which were mild to moderate; no serious AEs (SAEs) occurred. In 50, 70 and 100 mg groups there were mild to moderate increases in heart rate and systolic blood pressure that significantly correlated with higher levels of IGF1. TV-1106 showed pharmacokinetic characteristics of a long-acting hGH as demonstrated by a terminal elimination half-life of 23–35 h, delayed time of peak concentration, and systemic levels seen up to 7 days after dosing. IGF1 levels increased in a dose-dependent manner, before reaching a plateau, with levels above baseline extending beyond 7 days post dose.

**Conclusion:**

Single administration of TV-1106 up to 100 mg was safe in healthy volunteers. Pharmacokinetics and pharmacodynamics support once-weekly administration in patients with GHD.

## Introduction

Growth hormone (GH) promotes linear growth by regulating the endocrine and paracrine production of insulin-like growth factor 1 (IGF1), which is produced by the liver and other target tissues, such as the epiphyseal growth plate. GH's diverse metabolic actions also include anabolic, lipolytic and diabetogenic effects (1). Recombinant human GH (hGH) is primarily used to replace GH action in hypopituitary patients with GH deficiency (GHD): in children, to promote GH-dependent growth, and in adults, to correct GHD-induced metabolic abnormalities. To exert these effects over the long term, hGH has to be administered by daily s.c. injections (2). Studies on compliance in patients with GHD have shown that ∼25% of patients missed more than two injections per week and that difficulties with injections may contribute to this lack of compliance (3, 4, 5). Long-acting GH preparations have been studied before and have shown reduced injection frequency (6, 7).

TV-1106 is being developed by Teva Pharmaceuticals, Ltd as a long-acting hGH treatment for both adult and pediatric GHD. TV-1106 is a hGH preparation composed of mature human serum albumin (HSA) fused at its C-terminus to the N-terminus of mature hGH. Previous studies have shown that therapeutic proteins when fused to HSA have a longer half-life and decreased plasma clearance (8, 9). Based on this, it is anticipated that TV-1106 treatment will have a longer half-life and sustained exposure, thus, as a therapeutic agent for GHD, it will enable patients to reduce the number and frequency of injections, hopefully improving compliance and quality of life.

TV-1106 development was originated by Human Genome Sciences (HGS, Rockville, Maryland). In a phase 1/2 study of 42 patients, TV-1106 was found to be safe and well tolerated at single doses of 10, 30, 60 μg/kg or two doses of 60 μg/kg administered 1 week apart (TV-1106 Investigator Brochure, data on file Teva Pharmaceuticals).

Teva improved the manufacturing of the product in terms of yield and product purity, and this new formulation has previously not been studied in humans. The first dose for the present study was based on the principles of FDA guidance (10) and derived from two studies carried out in cynomolgus monkeys that determined the appropriate starting dose of 3.0 mg (the lower of two maximum recommended starting doses (MRSD): from the no observed adverse event level (NOAEL) MRSD was 39 mg and from pharmacology active dose (PAD) it was 3.0 mg) to a maximum dose of 100 mg, which was approximately fourfold lower than the human equivalent dose (HED) in cynomolgus monkeys (data on file, Teva Pharmaceuticals Study report 26331).

The primary objectives of this phase 1 study were to assess the safety, local tolerability, pharmacokinetics and pharmacodynamics of TV-1106 following single escalating s.c. doses ranging from 3 to 100 mg in healthy male volunteers.

## Subjects and methods

### Subjects

Eligible male subjects were between 20 and 40 years of age, with a BMI within the range of 19–30 kg/m^2^. Females were not eligible for the study because estrogen reduces the pharmacological activity of GH as measured in serum insulin-like growth factor (IGF1) levels (11). Subjects had to be in good health and have a serum IGF1 level within the normal range of −2.0 to +2.0 SDS (age-adjusted standard deviation scores). Exclusion criteria included known history of severe allergic or anaphylactic reactions, known allergy or hypersensitivity to recombinant GH or HSA, and a history of any clinically significant medical conditions (i.e., cardiac, endocrine, hematological, hepatic, renal, metabolic, neurological or other disease). The trial was conducted in accordance with the Declaration of Helsinki and ethical principles of Good Clinical Practice, according to the ICH Harmonized Tripartite Guideline; all subjects provided written informed consent.

### Study design

Study was a double-blind, placebo-controlled single ascending dose trial in healthy volunteers. Subjects were assigned to one of seven dose groups (3, 10, 25, 35, 50, 70 and 100 mg); within each dose group of eight subjects, six subjects received a single dose of TV-1106 and two subjects received a single dose of placebo by s.c. injection to the arm. There was at least a 7-day interval between two consecutive dose groups to ensure that the safety and tolerability of the previous dose levels were acceptable to the principal investigator and the sponsor before moving to a higher-dose group.

Subjects remained at the study clinic from the day prior to dose administration (Day −1) to 72 h after dosing (Day 4). Subjects returned to the clinic for safety assessments, pharmacokinetic (PK) and pharmacodynamic (PD) sampling on Days 5, 6, 8, 10, 12 and 15 and were asked to come back to the clinic for a follow-up visit 4 weeks after dosing (Day 29).

### Treatments

TV-1106 was administered using single-use vials. Each vial contained 25 mg/ml TV-1106 in 10 mM sodium phosphate, 200 mM mannitol, 60 mM trehalose dehydrate, 0.08% (w/v) polysorbate 80, pH 7.2. The placebo consisted of the formulation buffer without TV-1106. Subjects in the 3, 10 and 25 mg dose groups received one s.c. injection of 1 ml to the left arm using increasing TV-1106 concentrations or placebo. Subjects in the 35 and 50 mg dose groups received one 1 ml s.c. injection per arm using a 25 mg/ml TV-1106 solution or placebo. Subjects in the 70 and 100 mg dose groups received two s.c. injections of 1 ml each, per arm using a 25 mg/ml TV-1106 solution or placebo.

### Safety assessments

Subjects were asked about the occurrence of adverse effects (AEs) throughout the study period. During the clinic visits, safety electrocardiograms (ECGs), vital signs, laboratory assessments and fundoscopy were conducted, as well as injection site examinations by the investigator or clinic staff.

### Immunogenicity

Antibodies against TV-1106 were evaluated at check-in (Day −1), Day 15 and Day 29. Subjects with a positive immunogenicity sample post-dosing were requested to provide another sample ∼3–5 months after dosing. Analysis for anti-drug antibodies (ADA) was performed by Teva Biopharmaceuticals USA (Rockville, Maryland, USA) by following a multi-tier approach. Serum samples were first screened and confirmed for the presence of ADA using validated ELISA methods. Any samples tested above the screening and confirmatory cut-points were consider ADA-positive and confirmed ADA-positive, and were further characterized to evaluate the titer and neutralizing activity of the ADA using validated titration ELISA and neutralizing cell based assays respectively.

### ECGs and extensive ECG analysis

Standard (singleton) 12 lead ECGs were reviewed for safety by the site investigator and were conducted at the following time points: screening, pre-dose, 4, 12, 24, 48, 72, 168, 336 h after dosing and at the follow-up on Day 29.

The effect of TV-1106 on cardiac repolarization, conduction, rhythm and ECG morphology was also investigated in a dedicated comprehensive analysis. ECGs for centralized analysis were collected digitally and read by a core ECG laboratory (ERT, Philadelphia, PA, USA). ECGs for central analysis were collected at the following time points: sextuplicate ECGs within 60 min prior to dose on Day 1 as baseline, triplicate ECGs at 2, 4, 8, 12, 24, 48, 72 h after dosing, singleton ECG at 168 and 336 h after dosing.

### Serum pharmacokinetic parameters

Blood samples for PK analyses were collected within 60 min prior to dosing, and at 1, 2, 4, 6, 8, 12, 18, 24, 30, 36, 48, 60, 72, 96, 120, 168, 216, 264 and 336 h post-dose. Analysis of drug concentrations in serum of TV-1106 were performed by Teva Biopharmaceuticals USA using a sandwich-validated ELISA method.

The principal PK parameters calculated by non-compartmental analysis included: maximum observed concentration (C_max_), time of observed maximum concentration (T_max_), area under the concentration-time curve from 0 to last measurable concentration (AUC_0–t_), area under the concentration-time curve from 0 to 168 h post dose (AUC_0–168_), area under the concentration-time curve from 0 extrapolated to infinity (AUC_0–inf_), terminal half-life (t_1/2_), apparent total body clearance (CL/F) and the apparent volume of distribution (V_z_/F). Dose-normalized PK parameters were calculated and dose proportionality was examined.

### Pharmacodynamics

Blood samples for IGF1 were collected at screening, check-in (Day −1), at ∼18 and 12 h prior to dosing, pre-dose (within 60 min prior to dosing), 2, 4, 8, 12, 18, 24, 30, 36, 48, 60, 72, 96, 120, 168, 216, 264, 336 h post-dose and at Day 29 follow-up. Blood samples for the IGF binding protein-3 (IGFBP3) were also collected at pre-dose (within 60 min prior to dosing), 24, 48, 72, 96, 120, 168 and 336 h postdose.

Serum samples for IGF1 and IGFBP3 were analyzed by Quest Diagnostics (Wood Dale, IL, USA) using validated liquid chromatography/mass spectrometry–mass spectrometry (LC/MS–MS) and immunochemiluminescence assays respectively. The following PD parameters were calculated where applicable for both IGF1 and IGFBP3: maximum observed response (E_max_), time of observed maximum concentration (TE_max_), area under the effect-time curve from 0 h to the time of the last quantifiable measure (AUEC_0–t_), and area under the effect-time curve from 0 to 168 h post dose (AUEC_0–168_).

### Statistical analysis

Safety data were summarized using descriptive statistics and change from baseline (where appropriate) for each treatment arm. Data from the placebo subjects in the seven cohorts was pooled. Pharmacokinetic analyses for TV-1106 were performed using standard noncompartmental approaches (12) with SAS Version 9.1.3. and WinNonlin Version 5.3. Dose proportionality was examined for ln-AUC_0–t_, ln-AUC_0–inf_ and ln-C_max_ using the model (13) Ln (X)=C+β×ln (dose) where C is a constant, Ln (X) is the natural log transformed PK parameter and β is the population slope. A point estimate of β and 90% CI was determined, and dose proportionality was to be concluded if the 90% CI for the slope was completely contained in the range of (0.80, 1.25).

The PD parameters for IGF1 and IGFBP3 were calculated using standard noncompartmental approaches with SAS Version 9.1.3. Results were presented unadjusted and adjusted for baseline. The adjustment for baseline was subject-specific by subtracting the average baseline concentration for IGF1 (−18, −12, 0-h) from post-dose concentrations. Negative values obtained as a result of adjusting the data were kept negative for PD analysis and were not artificially adjusted to zero. A similar adjustment method was used for IGFBP3 except that only one value at 0 h was measured. For IGF1, results were presented in mass/volume as well as in SDS. IGF1 SDS reference ranges are age-dependent. The model for the adult reference range was developed by Quest (14).

#### Extensive ECG analysis

The baseline for the ECG analyses was the mean value of the six pre-dose ECGs on Day 1. For each time point, ECG interval data (heart rate (HR), RR, QT, PR and QRS) were averaged from all available replicates. Likewise, HR-corrected QT values (QTc) were derived from the average RR and QT values of the replicate ECGs of each time point. The QT interval was corrected for HR using the Fridericia correction (QTcF=QT/RR^1/3^) (15). Upper and lower 95% CIs were calculated for the changes from baseline. Comparisons of individual dose levels with placebo were made using an analysis of covariance, with factors for dose and subject, and using the relevant baseline value as covariate. The slope and *P* value for a linear regression on dose and a *P* value for non-linearity were derived. QTc values exceeding 450 ms, 480 ms or 500 ms and changes from the study baseline >30 ms or >60 ms were assessed for outlier analysis.

The relationship between TV-1106 plasma concentration and the change in (baseline and placebo adjusted) QTcF interval was assessed using a random intercept mixed-effects linear regression model. The predicted QTcF prolongations at the mean C_max_ values for each dose level, together with their upper (one-sided) 95% confidence limits were also produced.

#### Exploratory analysis of vital signs vs IGF1 levels

In addition to the analysis predefined above, an exploratory analysis was performed in order to identify any possible association between HR and blood pressure (diastolic and systolic) with the IGF1 concentrations. Four potentially explanatory variables were assessed: diastolic blood pressure (DBP), systolic blood pressure (SBP), body temperature and the IGF1 concentration in the blood. Each of the explanatory variables was analyzed in a similar way to the analyses of the ECG data described above. The relationship between HR and each of the potentially explanatory variables was assessed using individual (i.e., by subject) linear regressions and presented by dose level.

Finally, PK/PD analyses, using a random intercept mixed model, similar to that used for the ECG analysis, were performed for the following variables (using changes from baseline): HR vs IGF1; SBP vs IGF1; HR vs SBP; HR vs SBP and IGF1; and QTcF vs IGF1, as the dependent and independent variables respectively.

## Results

### Subject disposition and demography

A total of 56 subjects participated in the study, 42 received TV-1106 and 14 received placebo. One subject in the 35 mg cohort discontinued prematurely during the study due to non-compliance after receiving his dose. All other subjects completed the study as per protocol. The demographic characteristics of the dose groups were comparable (Table 1).

### Safety

Eighteen out of 56 healthy volunteers reported 43 adverse events that were mild to moderate in intensity. Overall, the most common AE (reported by two or more subjects) were increases in liver enzymes alanine aminotransferase (ALT) or aspartate aminotransferase (AST), cough, headache, hyperglycemia, injection site pain, nasopharyngitis, oropharyngeal pain and rhinorrhea. 40% of AEs were indicated by investigator as having no possible relationship to study treatment (Supplementary Table 1, see section on supplementary data given at the end of this article). The AEs appear to be dose-dependent as there was an increase in the number of AEs in the 70 and 100 mg dose groups (four out of six subjects, and five out of six respectively) compared to the lower doses (two out of six subjects for doses 3, 25 and 50 mg groups; no AEs in the 10 and 35 mg groups). All changes in laboratory measures over the course of the study were unremarkable or returned to normal prior to the end of the study. Three subjects (at the high dose level) had laboratory parameters considered clinically significant. Of them, one subject had significant increases in ALT, AST and glucose, one subject had increases in glucose, which were deemed as possibly related to study treatment, and one subject had increases in ALT and AST, which was not deemed as possibly related to study treatment. No serious AEs (SAEs) or deaths occurred during the study. One subject reported pain in the 35 mg group and two subjects in the 100 mg group reported tenderness at injection site. All reports were considered mild in severity and there were no observations of induration or erythema. No subject withdrew from the study due to AEs.

There were no findings of clinical significance regarding safety ECGs, vital signs or physical examinations. Three subjects tested positive for ADA during post-dose sampling; however, no neutralizing activity was observed. At follow-up samples collected 3 months post-dose, they were negative on ADA.

### Cardiac safety assessment

Safety ECGs in general showed that mean values for ECG parameters had no changes of clinical relevance. Changes from baseline were observed for several doses and, in particular, the 100 mg dose for QTcF; however, the observed variability in all cohorts was considered normal physiologic variability and did not represent a clinically significant trend or abnormality. The majority of subjects had one or more ECG findings with abnormalities; nonetheless, no potential clinically significant ECGs results were found.

**Figure 1:**
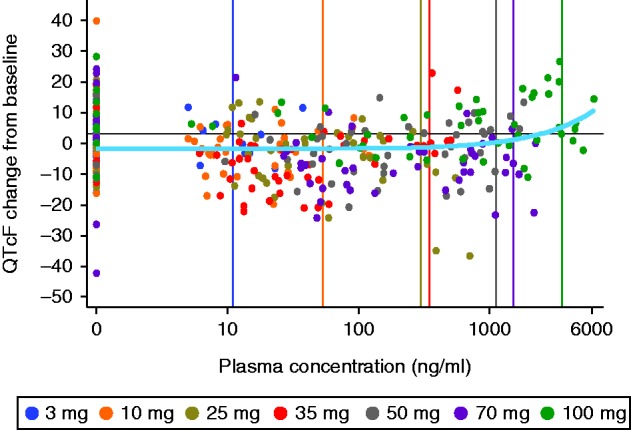
Extensive ECG results: PK/PD mixed-effects model for QTcF on plasma concentration. Vertical lines indicate mean C_max_ for each dose.

A dedicated analysis of the effect of TV-1106 administration on cardiac repolarization, conduction, rhythm and ECG morphology was performed. A rise in HR (as shown by a decreased ECG RR interval) was observed at TV-1106 doses of 50 and 100 mg, between 24 and 72 h post dosing. The effect was delayed relative to the peak plasma concentration of TV-1106, which occurred between 12 and 24 h. The QT interval, adjusted for HR using the Fridericia QT correction, showed decreases relative to baseline in all dose levels except for the 100 mg dose level (at 24 h post dose), which showed evidence of a significant QT prolongation, where the mean difference was 10.4 ms with the maximum upper limit of the 95% CI of 16.9 ms. The PR interval showed decreases of up to 10 ms at several dose levels at 48 and 72 h, reflecting similar reductions in the RR interval, while the QRS interval displayed no consistent effect of TV-1106. The PK/PD analysis indicated a shallow but statistically significant positive slope (0.00195 ms/ng per ml) between the plasma concentration of TV-1106 and changes in QTcF compared to baseline. However, the upper 95% one-sided limit for the predicted QTcF prolongation at the mean C_max_ concentration at the highest dose tested (100 mg 7.18 ms) is below the regulatory threshold of concern of 10 ms (Fig. 1). There was no evidence of an effect of TV-1106 on ECG rhythm, conduction or morphology.

The exploratory analysis assessing the relationship between HR, BP and IGF1 levels suggested that higher doses of TV-1106 may indirectly affect HR and BP in healthy subjects by increasing the circulating levels of IGF1, which in turn increases both HR and SBP. Nevertheless, this analysis provided no evidence for an association of QTcF with IGF1, further supporting the primary PK:PD analysis.

### Pharmacokinetic

Pharmacokinetic results at the 3 mg dose were considered unreliable since the lower limit of quantification (LLoQ) of the analytical method were above 5% of C_max_ for most subjects. Considerable variability was observed in the concentration values within and between cohorts. In addition, the K_el_ could not be determined for some subjects in the 3.0, 25 and 35 mg groups; consequently, the PK values for AUC_inf_, t_1/2_, CL/F and V_z_/F could not be calculated for all members of these treatment groups.

**Figure 2:**
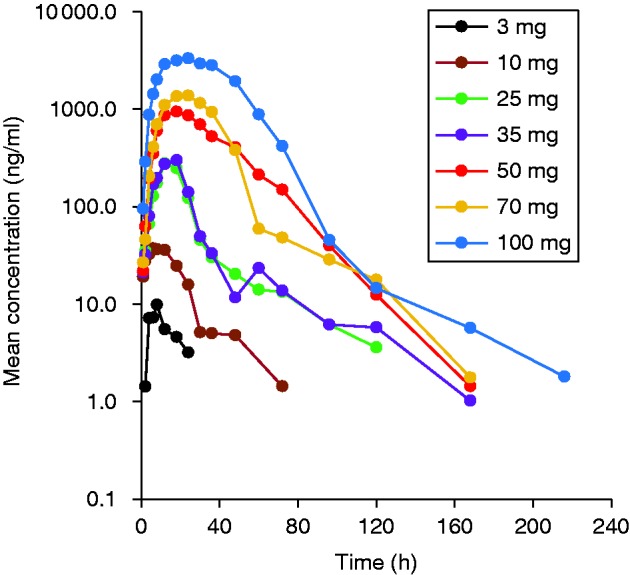
Mean serum concentrations of TV-1106 in semi-log scale.

In general, C_max_ and AUCs appeared to increase more than proportionally with increasing doses, bearing in mind, though, that at low doses concentrations were often below the limit of quantification (BLQ) and therefore the AUC was underestimated. Concentration values were detectable to ∼120 h with only six subjects having concentration values at 168 h and one subject up to 216 h (Fig. 2). At the lower doses (3–50 mg), with increasing doses, a delay in the median T_max_ was observed from 6 to 21 h (Table 2). T_max_ was consistent at higher doses (50–100 mg) where the median T_max_ occurred at 21 h. No specific trend was observed with increasing doses for apparent terminal half-life (t_1/2_) and remained fairly constant (ranging from 23.17 to 35.57 h), suggesting that the apparent terminal half-life is in agreement with a long half-life drug. A high inter-subject variability was also observed for the mean apparent total body clearance and the apparent volume of distribution within each cohort. However, at high doses (50–100 mg), and consequently with an increase in AUCs, the mean apparent total body clearance and apparent volume of distribution decreased approximately by half from 2.2 to 1.1 l/h and 96.5 to 46.1 l respectively. This was expected, as a more than dose-proportional increase was observed in AUC as previously described.

### IGF1 and IGFBP3 pharmacodynamic

#### IGF1

**Figure 3:**
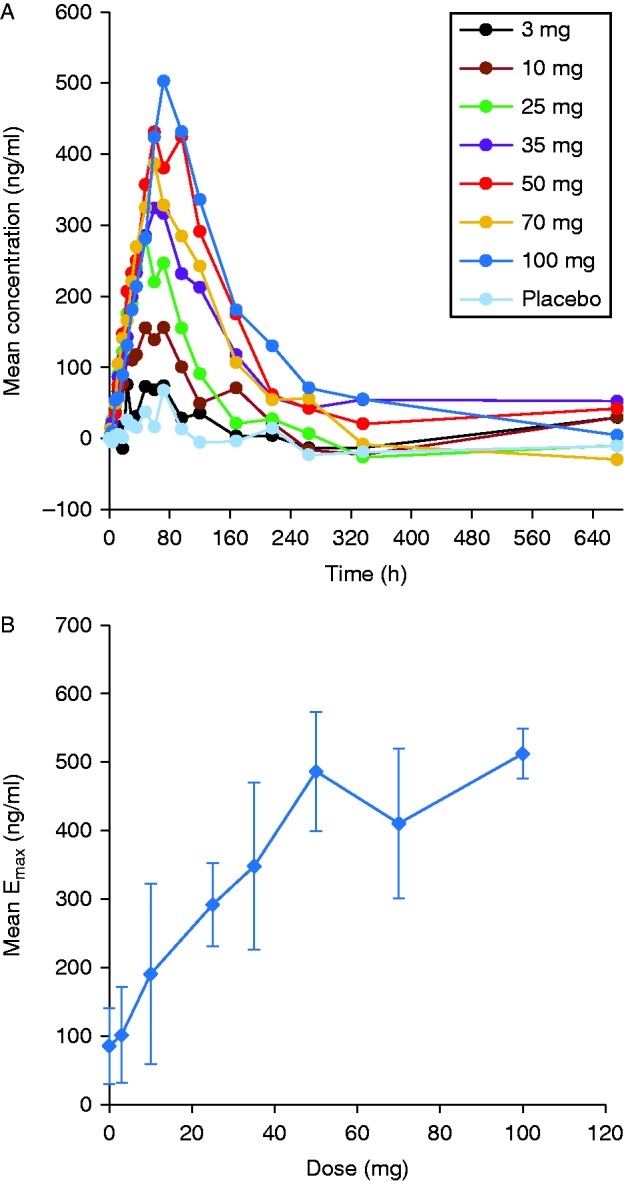
(A) Mean serum concentrations of IGF1. (B) Mean IGF1 maximum observed response (E_max_) in relation to TV-1106 dose. IGF1 concentrations in panels A and B were adjusted for each subject by subtracting the baseline concentration calculated from a mean of pre-dose concentrations.

Mean serum IGF1 levels prior to dosing with TV-1106 were ∼200 ng/ml. IGF1 levels increased rapidly to above baseline within ∼12–24 h for most subjects including placebo. Minimal to no difference in the IGF1 response vs placebo was seen at the lowest dose of 3 mg. Interestingly, there was a slight increase in IGF1 levels in the placebo group that could be attributed to a placebo effect (16). The maximum observed IGF1 response occurred between 48 and 72 h (Fig. 3a) and lasted for ∼168 h post dose (1 week). The relationship between the maximum observed response (E_max_) and doses of TV-1106 are shown in Fig. 3b. Overall, IGF1 E_max_ increased with dose and appeared to have reached a plateau between 50 and 100 mg. The overall effect (AUECs) was also dose-dependent and appeared to have plateaued at 35–100 mg (Table 3). The mean time to E_max_ remained fairly constant, ranging from 48 to 72 h across cohorts.

**Figure 4:**
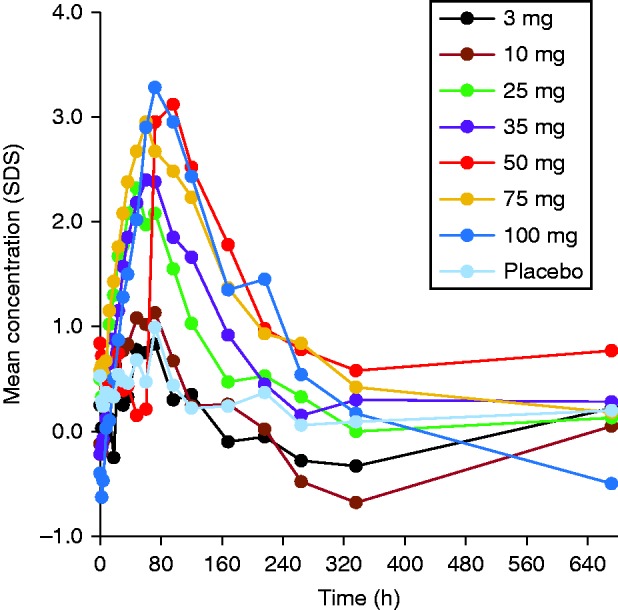
Mean serum concentrations of IGF1 in SDS unit (unadjusted).

The mean IGF1 SDS vs time profiles is presented in Fig. 4. When IGF1 concentrations were converted to SDS units, the 3 mg cohort showed values similar to the placebo cohort. A small but gradual increase was observed for E_max_ for doses ranging from 10 to 50 mg (Table 4). E_max_ values comparable to the 50 mg cohort were obtained for the 70 and 100 mg dose cohorts. TE_max_ remained fairly constant, ranging from 60 to 72 h, with the exception of the 3 and 25 mg dose groups (36 and 48 h respectively). When considering the maximum observed IGF1 (SDS) response (E_max_) in relation to the dose, E_max_ appeared to increase in an approximately linear fashion from 10 mg up to 50 mg where it reached a plateau. The 35 mg dose did not appear to show a difference from the 25 mg dose, but this may be attributable to high inter-subject variability observed within the 35 mg treatment group.

#### IGFBP3

**Figure 5:**
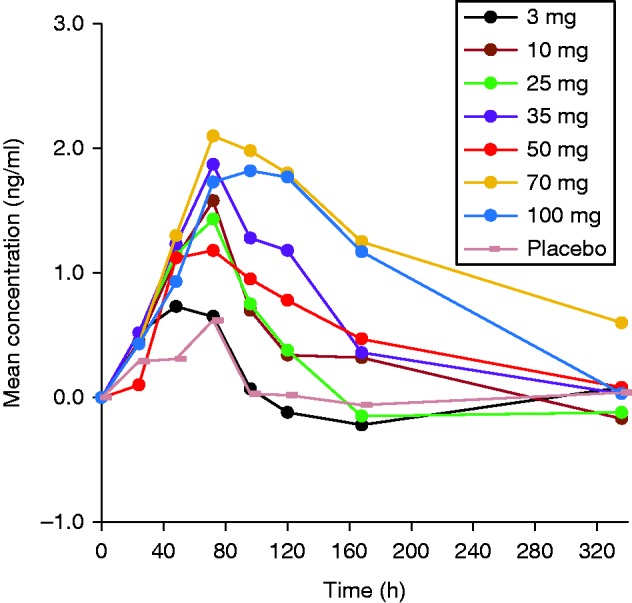
Mean serum concentrations of IGFBP3. IGFB3 concentrations were adjusted for each subject by subtracting the baseline concentration.

The mean baseline (pre-dose) IGFBP3 concentration value, pooled over all healthy volunteer subjects, was ∼4.2 mg/l. When the data were corrected for baseline, a small increase in IGFBP3 serum concentrations was observed in most subjects within ∼24 h following the administration of TV-1106 (Fig. 5). The TE_max_ remained fairly constant, occurring at ∼72 h for 3–50 mg dose cohorts (Table 5). For the higher doses the TE_max_ was longer and the median TE_max_ ranged between 84 and 96 h. After the TE_max_ IGFBP3 concentrations slowly decline toward returning to baseline values around 168 h. However, concentration values were still above baseline values around 168 h post dose in dose cohorts of 35 mg and over. The highest increase in AUEC_0–t_ and E_max_ occurred at the 70 mg dose, possibly showing that the maximum effect may have been reached.

## Discussion

TV-1106 is being developed to provide a long-acting GH therapy for patients with GHD. The dose range of this study started with the low dose of 3 mg up to a dose of 100 mg in a single s.c. injection in healthy volunteers. Based on clinical experience with daily rGH in children and adults, weight-based pediatric doses are usually three- to fivefold higher than weight-adjusted adult doses (adult doses are usually fixed) (17). As TV-1106 is being developed for both adult and pediatric patients with GHD, and single-dose safety data in healthy adults is pertinent to later-stage clinical development in both adults and children, incremental dose escalations to a dose of 100 mg were used to provide a safety margin for later development stages. Future studies will include GHD patients to evaluate safety, efficacy, PK and PD (IGF1).

The safety results of this study suggest that TV-1106 was well tolerated, AEs were mild to moderate in intensity and appeared to increase with increasing doses (70 and 100 mg). In contrast, it did not appear that the AEs reported in dose groups 3–50 mg were dose-dependent, as there was no pattern of increasing numbers of AEs reported with each subsequent dose group. Laboratory abnormalities reported to be clinically significant were seen only at 100 mg and included elevated liver enzymes and elevated glucose levels that returned to normal by the end of the study. Transient, non-neutralizing ADA at low frequency were observed in three TV-1106-treated subjects. Follow-up samples collected at ∼3–5 months post dosing revealed an absence of ADA. Reports of elevated glucose levels and transient ADA have been seen with other long-acting GH preparations (6, 7).

HR and SBP were shown to be elevated in some subjects receiving the 50–100 mg doses and a careful exploration of the possible factors related to these events was conducted. This exploratory analysis suggested that TV-1106 induce higher levels of IGF1 in healthy subjects, which, in turn, appears to be directly related to increase in HR and SBP. The extensive QT/QTc analysis conducted accordingly with current regulatory and scientific standards supports the favorable cardiac safety profile of TV-1106 at dose levels up to and including 50 mg, as meeting the current regulatory safety standards for QT/QTc interval prolongation (18). The QT/QTc prolongation effect of TV-1106 was limited to the highest dose (100 mg) tested in TV-1106-SAD-102 study, and was not supported by the PK/PD analysis. Importantly, cardiovascular effects observed at IGF1 levels of up to +3.5 SDS in healthy volunteers with normal physiologic baseline levels of GH who are administered exogenous GH, may not be relevant to GH deficient patients where the therapeutic goal is to restore a normal physiologic GH level (represented by IGF1 of −2 SDS to +2 SDS).

The pharmacokinetic results of this phase 1 study suggest that TV-1106 has characteristics of a long-acting GH formulation with delayed peak concentration times, a long half-life and systemic exposure over ∼7 days. The PK properties of TV-1106 support its use as a long-acting GH product and suggest that a reduced once-a-week frequency of injections (compared to daily injections) may be possible. Duration of the half-life of TV-1106 is in agreement with that seen with other long-acting GH drugs. (6, 19, 20, 21, 22).

The current study only dosed male subjects, so it is therefore not possible to predict the impact of gender on TV-1106's concentration-time profile. In general, TV-1106 concentrations showed an increase with doses ranging from 3 to 100 mg with non-dose proportional increase. This is consistent with the more-than-dose proportionality detected using the ANOVA model.

The therapeutic effects of GH are mediated by IGF1. IGFBPs play important regulatory roles in controlling the bioavailability and action of IGF1, and are regulated by various factors including GH. About 90% of IGF1 is bound to IGFBP3. Because hGH preparations induce significant increases in IGF1 and IGFBP3 related to GH mechanism of action, these molecules are accepted as PD measures for GH activity; however, the spurious relationship between serum IGF1 levels and growth response precludes the use of IGF1 as a surrogate marker for efficacy (17). Both IGF1 and IGFBP3 showed increases in concentrations with a maximum observed response occurring at approximately at 2–3 days and 3–4 days respectively, and the effects lasting for ∼7 days. The results of these surrogate markers also showed that dosing administration for TV-1106 could be less frequent than the currently required daily injection with rhGH. There was a dose-dependent increase in IGF1 with the maximum observed mean E_max_ and AUEC occurring with the 100 mg dose. However, the 100 mg dose had only slightly higher results than that of the 50 mg dose and the 70 mg dose had lower mean values, so there may be a plateau of effect beginning at the 50 mg dose. It seems that there is an indirect effect between TV-1106 and IGF1; peak IGF1 was 2–3 days post dose, as TV-1106 maximum concentration was achieved within 21 h. In addition TV-1106 increased more than dose proportionality but IGF1 reached a plateau between 50 and 100 mg.

The increases in IGF1 levels for TV-1106 are consistent with the pre-clinical and clinical evaluation previously found for TV-1106 formulation developed by HGS (Rockville, Maryland). The earlier phase 1/2 study by HGS conducted with patients with adult onset GHD found an increase in IGF1 levels after administration of TV-1106 (early stage of development) with a peak around 2–3 days and a slow decline lasting for up to 7 days (data on file, Teva Pharmaceuticals).

Currently, patients with GHD are required to administer daily injections of recombinant GH, and this has led to problems in compliance for some patients. The poor compliance in turn has been shown to result in decreased growth responses in patients (4). The results of this single-dose study suggest that TV-1106 appears as a long-acting hGH preparation that may be able to reduce the number and frequency of injections in the treatment of GHD, and future development of this drug should be considered. In continuation of TV-1106 drug development, two phase 2 studies, one in adults and one in pediatric GHD populations, have been initiated.

## Supplementary data

This is linked to the online version of the paper at http://dx.doi.org/10.1530/EJE-15-0554.

## Figures and Tables

**Table 1:** Subject demographics. Data are presented as mean (s.d.)

**Dose**	***n***	**Age** (years)	**Height** (cm)	**Weight** (kg)	**BMI** (kg/m^2^)	**Race** Number of subjects
3 mg	6	31.2 (3.4)	177 (6.5)	72 (6.9)	23 (2.7)	Asian 1 Black 2 White 3
10 mg	6	22.5 (2.2)	176 (6.6)	76 (8.9)	24 (3.3)	Asian 2 Black 0 White 4
25 mg	6	24.2 (4.3)	179 (6.5)	83 (10.1)	26 (1.5)	Asian 0 Black 0 White 6
35 mg	6	26.0 (3.8)	176 (7.1)	83 (8.1)	27 (0.9)	Asian 0 Black 1 White 5
50 mg	6	24.3 (3.3)	181 (4.6)	82 (10.9)	25 (2.6)	Asian 0 Black 1 White 5
70 mg	6	23.7 (3.7)	180 (2.4)	80 (11.2)	25 (3.7)	Asian 1 Black 2 White 3
100 mg	6	28.0 (6.8)	175 (4.1)	82 (9.0)	27 (2.3)	Asian 1 Black 0 White 5
Pooled placebo	14	29.4 (6.1)	178 (5.2)	83 (11.9)	26 (2.8)	Asian 0 Black 2 White 12

**Table 2:** Pharmacokinetic parameters of TV-1106.

**Dose**	**AUC_0–t_** (ng·h/ml) Mean (s.d.)	**AUC_0–inf_** (ng·h/ml) Mean (s.d.)	**AUC_0–168_** (ng·h/ml) Mean (s.d.)	**C_max_** (ng/ml) Mean (s.d.)	**T_max_** (h) Median (range)	**T_1/2_** (h) Mean (s.d.)	**CL/F** (l/h) Mean (s.d.)	**V_z_/F** (l) Mean (s.d.)
3 mg^a^ (*n*=2)	258.9 (182.6)	NA	NA	25.2 (16.1)	6.0 (4.0–8.0)	NA	NA	NA
10 mg (*n*=6)	878.9 (662.4)	1273.6 (713.4)	NA	53.6 (48.5)^b^	7.0 (1.0–18.0)	35.6 (14.4)^b^	10.5 (6.4)^b^	628.3 (609.7)^b^
25 mg (*n*=6)	5745.3 (4534.3)	12 308.4 (NA)	NA	298.4 (279.4)	18.0 (12.0–72.0)	26.6 (NA)^c^	2.0 (NA)^c^	77.9 (NA)^c^
35 mg (*n*=6)	6443.4 (5061.6)	6354.5 (3757.8)^a^	3360.9 (NA)^c^	347.9 (353.9)	10.0 (4.0–18.0)	31.0 (20.8)	6.7 (3.9)	357.9 (376.8)
50 mg (*n*=6)	37 357.1 (23 101.2)	38 395.5 (25 596.9)	64 752.0 (NA)	1117.6 (594.3)	21.0 (12.0–48.0)	27.1 (13.9)^d^	2.2 (2.1)^d^	96.5 (99.4)^d^
70 mg (*n*=6)	46 346.8 (23 876.6)	46 861.8 (23 942.1)	54 237.4 (NA)	1524.5 (613.1)	21.1 (12.0–30.0)	28.6 (4.35)	2.1 (1.5)	85.8 (60.3)
100 mg (*n*=6)	144 701.2 (80 065.0)	144 985.5 (79 945.8)	91 284.2 (60 937.0)	3542.7 (1972.9)	21.0 (12.0–72.0)	23.2 (16.3)	1.1 (1.0)	46.1 (57.9)

NA is used to represent results that could not be calculated.

a Only two subjects were included in the PK population as they had at least three concentrations above LLoQ. The mean (s.d.) descriptive statistics should be interpreted with caution.

b *n*=4.

c *n*=1 descriptive statistics (mean, s.d.) should be interpreted with caution.

d *n*=5.

**Table 3:** Baseline-adjusted PD parameters of IGF1.

**Dose**	**AUEC_0–t_** (ng·h/ml) Mean (s.d .)	**AUEC_0–168_** (ng·h/ml) Mean (s.d.)	**E_max_** (ng/ml) Mean (s.d.)	**TE_max_** (h) Median (range)
3 mg (*n*=6)	7774.1 (20 572.1)	5984.1 (5651.4)	101.9 (55.5)	48.0 (24.0–672.0)
10 mg (*n*=6)	17 259.3 (11 204.4)	14 933.3 (4883.6)^b^	190.9 (69.6)	72.0 (36.0–168.0)
25 mg (*n*=6)	19 471.2 (27 784.3)	24 173.2 (11 607.9)	292.1 (131.9)	54.0 (48.0–72.0)
35 mg (*n*=6)	65 252.2 (23 967.8)	34 616.1 (6755.8)^b^	348.2 (60.7)	66.0 (60.0–72.0)
50 mg (*n*=6)	71 159.33 (29 808.87)	47 508.0 (10 400.6)	486.4 (121.8)	66.0 (60.0–96.0)
70 mg (*n*=6)	40 340.99 (21 444.86)	39 159.0 (7641.0)	410.7 (86.7)	60.0 (48.0–72.1)
100 mg (*n*=6)	76 237.08 (18 132.73)	49 163.1 (7866.7)	512.1 (109.2)	72.0 (72.0–96.0)
Pooled placebo (*n*=14)	−4103.36^a^ (22 669.7)	2358.1 (4421.3)	85.7 (36.4)	72.0 (48.0–672.0)

a Concentrations dropped below 0 after 168 h, results should be interpreted with caution.

b *n*=5.

**Table 4:** Summary of IGF1 SDS mean pharmacodynamic parameters (unadjusted).

**Dose**	**IGF1 (SDS) at 0 h** Mean (s.d.)	**E_max_ (SDS)** Mean (s.d.)	**TE_max_** (h) Median (range)
3 mg (*n*=6)	0.2 (1.1)	1.1 (1.0)	36.0 (24.0–72.0)
10 mg (*n*=6)	−0.1 (0.6)	1.4 (0.6)	66.0 (36.0–168.0)
25 mg (*n*=6)	0.5 (0.7)	2.4 (1.0)	48.0 (36.0–72.0)
35 mg (*n*=6)	−0.2 (0.4)	2.6 (0.4)	66.0 (60.0–72.0)
50 mg (*n*=6)	0.4 (0.8)	3.4 (0.3)	66.0 (60.0–96.0)
70 mg (*n*=6)	0.6 (0.5)	3.0 (0.6)	60.0 (48.0–72.1)
100 mg (*n*=6)	−0.4 (1.0)	3.3 (0.9)	72.0 (72.0–96.0)
Pooled placebo (*n*=14)	0.5 (0.7)	1.2 (0.7)	72.0 (48.0–672.0)

**Table 5:** Baseline-adjusted PD parameters of IGFBP3.

**Dose**	**AUEC_0–t_** (mg·h/l) Mean (s.d.)	**AUEC_0–168_** (mg·h/l) Mean (s.d.)	**E_max_** (m/l) Mean (s.d.)	**TE_max_** (h) Median (range)
3 mg (*n*=6)	26.6 (116.9)	37.8 (47.0)	0.8 (0.3)	48.0 (24.0–72.0)
10 mg (*n*=6)	136.8 (140.3)	105.8 (42.6)^a^	1.6 (42.6)	72.0 (48.0–72.0)
25 mg (*n*=6)	81.8 (109.0)	104.2 (65.5)	1.5 (0.7)	60.0 (48.0–72.0)
35 mg (*n*=6)	202.5 (60.7)	168.7 (36.1)^a^	1.9 (0.2)	72.0 (72.0–72.0)
50 mg (*n*=6)	157.6 (161.2)	119.8 (91.1)	1.5 (0.7)	72.0 (48.0–120.0)
70 mg (*n*=6)	389.8 (255.8)	234.4 (120.0)	2.3 (1.0)	84.1 (72.0–168.0)
100 mg (*n*=6)	310.4 (149.5)	209.6 (92.5)	2.0 (0.7)	96.0 (72.0–120.0)
Pooled placebo (*n*=14)	24.4 (191.1)	29.2 (92.8)	0.8 (0.7)	72.0 (24.0–336.0)

a *n*=5.
